# O.4.5-5 Are more active children healthier in the long run? – Results from the MoMo study

**DOI:** 10.1093/eurpub/ckad133.213

**Published:** 2023-09-11

**Authors:** Simon Kolb, Alexander Burchartz, Leon Klos, Claudia Niessner, Carmen Volk, Birte von Haaren-Mack, Steffen Schmidt, Lara Tschuschke, Elena Schlag, Alexander Woll

**Affiliations:** Karlsruhe Institute of Technology (KIT), Germany; simon.kolb@kit.edu; Karlsruhe Institute of Technology (KIT), Germany; simon.kolb@kit.edu; Karlsruhe Institute of Technology (KIT), Germany; simon.kolb@kit.edu; Karlsruhe Institute of Technology (KIT), Germany; simon.kolb@kit.edu; Karlsruhe Institute of Technology (KIT), Germany; simon.kolb@kit.edu; Karlsruhe Institute of Technology (KIT), Germany; simon.kolb@kit.edu; Karlsruhe Institute of Technology (KIT), Germany; simon.kolb@kit.edu; Karlsruhe Institute of Technology (KIT), Germany; simon.kolb@kit.edu; Karlsruhe Institute of Technology (KIT), Germany; simon.kolb@kit.edu; Karlsruhe Institute of Technology (KIT), Germany; simon.kolb@kit.edu

## Abstract

**Purpose:**

Various recommendations emphasize the importance of regular physical activity (PA) for building and maintaining health in adults as well as in children and adolescents. Using longitudinal data from the German MoMo study, this analysis aimed at investigating whether children who were more active over time were healthier eleven years later compared to those that were less active over the time.

**Methods:**

1407 participants (55.5 % female; mean age at Baseline = 8.2 ± 3.8 y.) completed three times of measurement within three survey waves of the MoMo study (Baseline: 2003 – 2006; Wave 1: 2009 – 2012; Wave 2: 2015 - 2017). The study included a self-report questionnaire among others. Participants who reported active participation at all times of measurement were compared to those who reported no participation at all. The groups were compared regarding their self-reported health status, and whether they reported recurrent headache in the past three months. 95%-Confidence Intervals (95%CI) were used, and odds ratios were calculated to determine differences between the groups.

**Results:**

The active group was more likely to rate their health as “very good” (46.9%, 95%CI = [30.9,63.6]) compared to their fewer active peers (18.4%, 95%CI = [11.3,28.6]) (OR(95%CI) = 3.9 [1.6,9.7]). Additionally, the active group reported less recurrent headache (35.7%; 95%CI = [31.3,40.4]) at the last measurement point compared to the least active counterparts (55.5 %; 95%CI = [48.9,62.0]) (OR(95%CI) = .5[.3,.6]).

**Conclusions:**

Continuous physical activity during childhood and adolescence, e.g. in a sports club, not only increases the chance of a better self-perceived health, but also seems to reduce the risk of health impairments, such as recurrent headaches. Therefore, promoting physical activity as proposed by the World Health Organization and others is an important part of a healthy lifestyle.

**Support/Funding Source:**

This work has been developed within the MoMo-Study (MoMo) (2003 – 2021). MoMo is funded by the Federal Ministry of Education and Research (funding reference number: 01ER1503) within the research program ‘long-term studies’ in public health research.

